# Residents’ Diachronic Perception of the Impacts of Ecological Resettlement in a World Heritage Site

**DOI:** 10.3390/ijerph16193556

**Published:** 2019-09-23

**Authors:** Kai Wang, Menghan Wang, Chang Gan, Mihai Voda

**Affiliations:** 1 College of Tourism, Hunan Normal University, Changsha 410081, China; kingviry@hunnu.edu.cn (K.W.); linxirihan@gmail.com (M.W.); 1583086911gc@gmail.com (C.G.); 2 Faculty of Geography, Dimitrie Cantemir University, Targu Mures 545545, Romania

**Keywords:** ecological resettlement, residents’ diachronic perception, Wulingyuan scenic area

## Abstract

As one of the main factors in any tourist destination, residents’ perception of the impacts of ecological resettlement has a substantial influence on the sustainable development of any world heritage site. Our research takes the residents of three different resettlement locations in the Wulingyuan scenic area, a world heritage site, as the object of our survey. Based on questionnaire investigations in 2010 and 2016, this article analyzes the residents’ diachronic perception of the impacts of ecological resettlement. Independent sample *t*-tests and Analysis of Variance (ANOVA) are used to compare the differences in residents’ perception toward ecological relocation and analyse how demographic characteristics affect residents’ perception. Multiple stepwise regression analysis is applied to explore the main factors that contribute to the differences in the perception of impacts of ecological resettlement. The results show that during the study period, respondents have the strongest perceptions of the economic, socio-cultural, resource-environment and psychological impacts. However, they have negative perceptions of relocation policy impacts. Compared with 2010, residents with different gender, age, education level, income level and engagement in tourism have significant differences in perception of impacts of resettlement in 2016. Multiple stepwise regression analysis demonstrates that the perceptions of impacts of the ecological resettlement and economic policy are the primary factors to affect residents’ overall perceptions.

## 1. Introduction

Tourism has extensively been identified as a crucial sector of economic growth and development in world heritage sites [1,2]. Nevertheless, the phenomenon of over-commercialization resulted from the increasing demand for tourism consumption has become increasingly prominent, and, accordingly, the ecological environment faces serious challenges. In order to protect the authenticity of world heritage sites, China has implemented a series of ecological relocation projects [3]. Ecological relocation refers to population migration in order to protect or restore the particular ecology of a region [4]. The purpose of launching a resettlement is to seek harmony between humans and nature [5]. A community’s values are contributing to the local environment quality improvement, increasing the balance of all the geographical place components. Residents’ perception of the impacts of ecological relocation is crucial to ensure the success and harmony of world heritage sites. Consequently, it is of great significance to have insight into the dynamic change of the residents’ perception in world heritage sites.

The research, which has a bearing on residents’ perception of tourism began in the 1970s [6]. Thus far, scholars have devoted increasing attention to the rural tourism or leisure resorts in developed countries [7], and have failed to concentrate on tourism-based economic areas [8,9]. At the level of perception of the impacts of tourism, scholars mainly explored from the perspectives of economy [10,11,12,13,14,15], social culture [16,17,18,19,20,21] and ecological environment [22,23,24,25]. Some scholars asserted that the economic dependence of residents on tourism, the life cycle stage of tourism destinations and community dependence will affect residents’ perception of the impacts of tourism [16]. In addition, researchers have put forward that demographic characteristics may have influence on the residents’ attitudes toward tourism development, for instance, gender [26], age [27] and education level [28]; some scholars held that it is necessary to study the impacts of tourism [29,30]. At present, a larger number of perceptions of the impacts of tourism have been examined from contemporaneous and diachronic perspective. Regarding the diachronic comparison, Lee et al. [31] concluded that the opening of casinos had a more intensive influence on the residents’ quality of life than before. Contemporaneous comparison was carried out among others by Joan et al. [32], who compared the perceptions of impacts of tourism in Tenerife and Mallorca, two seaside resorts.

After Cowles’ proposed the concept of ecological resettlement, scholars from various countries have devoted more attention to its exploration. The impacts of ecological resettlement have become a crucial scope of the theoretical framework. In order to catch on the issue of ecological restoration of destinations, when the relocation planning has been implemented, some researchers have collected microbial information of environmental samples in national parks by pyrosequencing the DNA of snails [33]; Considering the impact of ecological resettlement, Cliggett [34] investigated residents who were obliged to relocate in the middle reaches of the Zambezi River, and deemed that the project had a profound influence on residents in terms of economy, politics and environment. When it comes to the evaluation of ecological resettlement performance, Xiang [35] held that ecological relocation promotes living standard, economic structure and ecological environment in the Guoluo Prefecture of Qinghai Province in China. While a number of researchers have placed a particular emphasis on the static investigation of a certain time section, scholars have failed to devote more attention to examine the residents’ diachronic perception towards ecological resettlement.

In order to bridge this gap, the cross-sectional research compares data collected in three representative resettlement locations, the Tianzishan community, the Gaoyun community and the Yuanjiajie community, in 2010 and in 2016. This study focuses on examining the residents’ diachronic perception of impacts of ecological resettlement in world heritage sites. The target of this research is to compare the changes in the residents’ perception of impacts of resettlement, and to advance understanding of how ecological resettlement affects the perception of the impacts and how it works in favor of tourism development. The findings of our research have prominent implications for local authorities responsible for the management of ecological resettlement in understanding the residents’ perception in support of the relocation. This study indicates that policymakers should dynamically supervise the performance of resettlement policies and improve the follow-up supportive measures. In the meantime, ecological relocations in the other world heritage sites, which optimize the resettlement policy and promote residents’ quality of life can take example from our findings.

The following chapters of this study are arranged as follows: The materials and methods are reported in Section 2, in which we present the process of ecological resettlement in the Wulingyuan scenic area, specify the research method, report the process and handle the collection of data. The empirical findings are presented in Section 3, in which we display the differences in residents’ perception of the impacts of ecological resettlement and compare the main factors. Section 4 discusses the findings, and the key conclusions are summarized in Section 5.

## 2. Materials and Methods

### 2.1. Study Area

**Figure 1:**
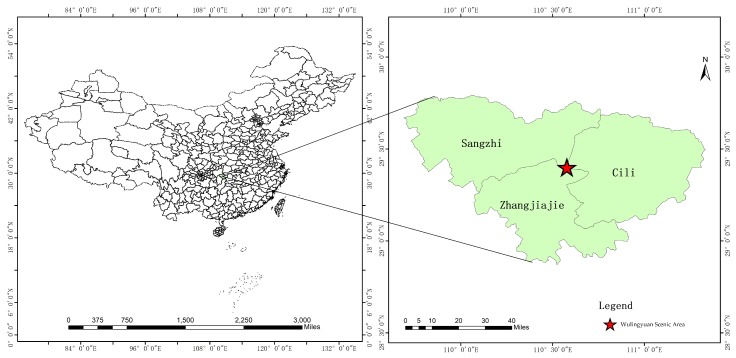
The location map of the study area.

The Wulingyuan scenic area (110°20′30″–110°41′15″E, 29°16′25″–29°24′25″N), a district of Zhangjiajie in China, was selected as the specific study case (Figure 1). It is located in the central Wuling Mountains of Hunan Province, and is comprised of four key scenic spots, including Zhangjiajie National Forest Park, Tianzi Mountain Nature Reserve, Suoxiyu Nature Reserve, and Yangjiajie Nature Reserve. Wulingyuan covers a total area of 397.58 km^2^, which are the first batches of World Natural Heritage and National Key Scenic Area in China. The unique natural values of global significance have brought opportunities for the rapid local tourism development. Notably, tourism has become a pillar industry of Wulingyuan, accounting for over 90% of its GDP in recent years.

In 1998, the United Nations Educational, Scientific and Cultural Organization (UNESCO) warned that the over-development of tourism and the serious urbanization tendency in Wulingyuan had caused irreversible environmental damage. From 1999 to 2001, local authorities demolished 124 reception facilities in the core attraction zone in two phases and resettled 1791 inhabitants. The first phase brought about the demolishment of 59 tourist reception facilities in Yuanjiajie scenic spot, Tianzishan scenic spot and other areas, the demolishment of 377 households and relocation of 1162 inhabitants. In the second phase, 65 tourist reception facilities were demolished in Huangshizhai scenic spot, Jinbianxi scenic spot and Yangjiajie scenic spot, and 629 inhabitants were relocated. In the recent years, the contradiction between heritage protection and tourism development in Wulingyuan has still been extremely prominent. Therefore, the administrative authorities have resolved to launch the third-phase of the ecological resettlement project, aiming at achieving sustainable development of the local tourism industry.

### 2.2. Research Methods

#### 2.2.1. Semi-Structured Interview

Interviewing is currently the most popular method for collecting data in phenomenological studies. There are various types of interviews, which can be divided into: structured interview, semi-structured interview and unstructured interview [36]. The semi-structured interview, which is the focus of this paper, provides a different method of information collection. Under this approach, the questions are somewhat structured, yet participants have the freedom to introduce new ideas during the interview. This could be the reason semi-structured interviews are considered one of the most effective and convenient ways of collecting qualitative scientific data. We visited the resettled families to find out the residents’ specific views on the implementation of ecological resettlement and the perception of impacts on ecological resettlement by filling in a total number of 703 questionnaires and interviews.

#### 2.2.2. Independent Sample *t*-Test

The independent sample *t*-test, which is the main non-invasive method, is applied to test the difference of data obtained by the two groups of non-correlated samples. This article compares and analyzes the differences of residents’ perception of the impacts of 34 indicators in 2010 and 2016, as well as the perception differences of demographic characteristics on ecological resettlement by means of the independent sample *t*-test.

#### 2.2.3. Exploratory Factor Analysis and ANOVA

Exploratory factor analysis is used to find the essential structure of multivariate observation variables and to deal with dimensionality reduction. The purpose of the exploratory factor analysis is to find out the potential structure of the scale and reduce the number of items. It identifies and groups those variables that provide redundant information about the variation in the data; these groups of variables constitute factors. Thus, exploratory factor analysis is an analysis of data derivation. Analysis of Variance (ANOVA) is applied to test the significance of the mean difference between two or more samples. Variance analysis starts with the variance of the observed variables and studies which variables of many control variables have significant influence on the observed variables. This article analyzes the perceived differences in demographic characteristics of ecological resettlement with ANOVA.

#### 2.2.4. Multiple Stepwise Regression Analysis

Multiple stepwise regression analysis is conducted to select and judge the indicators of residents’ overall perception of ecological resettlement in the relocated communities. The multiple stepwise regression equation is as follows:

*Y* = *b*_1_*X*_1_ + *b*_2_*X*_2_ + … + *b*_41_*X*_41_ + *e* (1)

where *Y* means “the overall satisfaction of respondents with ecological resettlement”; *X_1_–X_41_* respectively are seven demographic characteristics and 34 perception variables. In general, a variance inflation factor below 5.3 and a tolerance above 0.19 indicate that there is no multicollinearity among independent variables.

### 2.3. Index Selection of the Perception of Impacts of Ecological Resettlement

The local residents are farmers and there are minorities in the ethnic composition. The heritage site is about 35 km from their new locations. There were three main modes of resettlement: the residents of Tianzishan, represented by concealed resettlement, moved from the place where they were close to tourist attractions, such as Daguantai to Clove village; the respondents of Yuanjiajie, who relocated in an invest and develop relocation community, adopted the mode of operational aspects of hotels to run hotels and tourist shops; the residents of Gaoyun, represented by the unified resettlement, lived in separate places from commercial operations.

**Figure 2:**
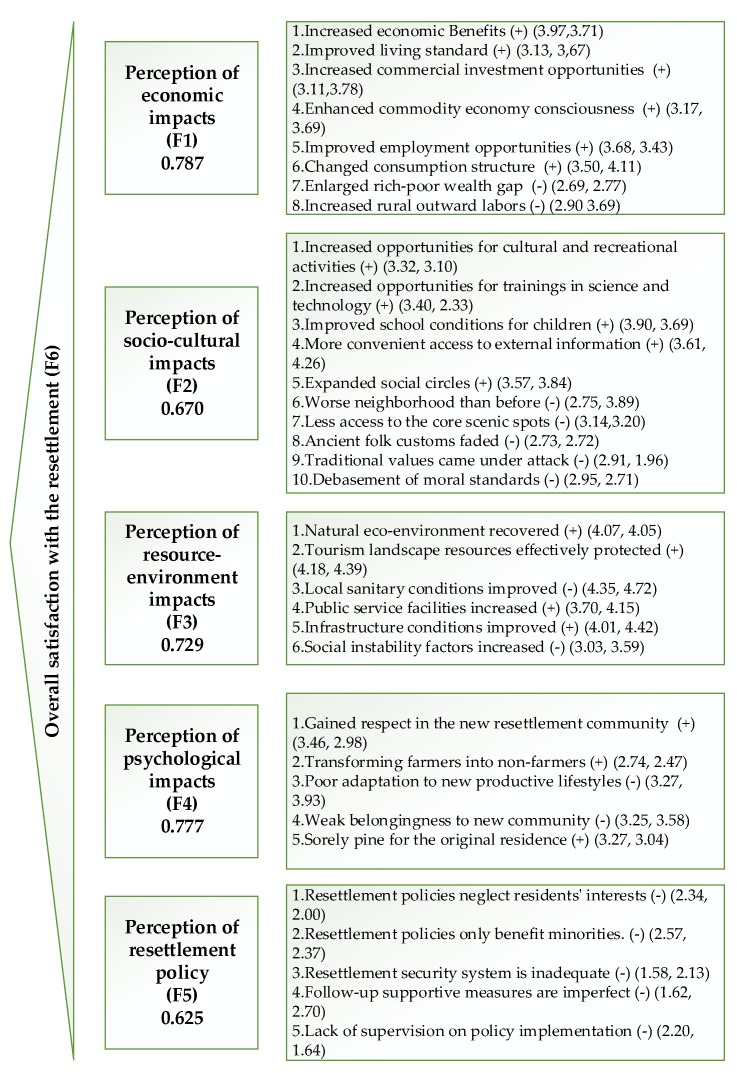
Evaluation framework and index system of resident’s perception of the impact of ecological resettlement. Note: the bold figures represent the Cronbach’s alpha coefficient of each index layer; (+) indicates a positive index, (−) indicates a negative index; (2.20, 1.64) the left side represents the 2010 index value, and the right side represents the 2016 index value.

The questionnaire was divided into two parts. The first part included 7 demographic characteristics, for instance, gender, age, monthly income, education level, engagement in tourism, proportion of tourism receipts and modes of resettlement. The second part listed 38 indicators of perception of impacts. The index system mainly referred to the relevant research results on the impacts of tourism and ecological resettlement [19,37,38,39,40,41,42,43]. Based on the local condition of relocation in Wulingyuan scenic area, our research completed the design with the assistance of listening to expert opinions. The positive indexes were based on the Likert 5 scale: 1 means “completely disagree”, 2 means “disagree”, 3 means “neutral”, 4 means “consent”, 5 means “completely agree”; nevertheless the negative indexes were based on the Likert 5 scale: 5 means “completely disagree”, 4 means “disagree”, 3 means “neutral”, 2 means “consent”, 1 means “completely agree”. From Figure 2 above, the paper has drawn a graphical in order to clearly show the directionality of every index.

### 2.4. Data Collection and Data Processing

Sampling was split across the three different resettlement locations in the Wulingyuan scenic area, namely Tianzishan community, Gaoyun community and Yuanjiajie community. Before the research team set out, they had received relevant training. Three hundred and sixty valid questionnaires correspond to the study area in 2010, among which 128, 121 and 111 valid questionnaires were collected in Tianzishan community, Gaoyun community and Yuanjiajie community, respectively. The 2016 research data was derived from the field survey conducted in the above three communities in April 2016. 343 valid questionnaires were obtained, and 115, 109 and 119 valid questionnaires were retrieved in Tianzishan community, Gaoyun community and Yuanjiajie community, respectively.

The Cronbach’s alpha result showed that the questionnaire had high reliability in 2010 (α = 0.837). The Kaiser-Meyer-Olkin (KMO) and Bartlett sphere test statistics indicated that the observation data were suitable for factor analysis (KMO and Bartlett statistics = 0.778). In our study, five common factors were extracted from 38 variables through exploratory factor analysis, 34 indicators were retained, and the cumulative variance contribution rate was 61.5%. The common factors were marked as “economic impacts”, “socio-cultural impacts”, “resource and environment impacts”, “psychological impacts” and “resettlement policy impacts”. On the basis of exploratory factor analysis, the Cronbach’s alpha coefficient of the overall questionnaire was 0. The Cronbach’s alpha coefficients of the five common factors were 0.787, 0.670, 0.729, 0.777 and 0.625 respectively, which demonstrated that the measurement items had preferable internal consistency.

## 3. Analysis and Findings

### 3.1. Demographic Characteristics of Two Surveys

The demographic characteristics of the two survey samples show that the proportion of male respondents in 2016 is slightly higher (51.9%) than in 2010 (48.6%). But on the whole, the proportion of male and female respondents is relatively balanced (Table 1). In terms of the age distribution, residents aged 21 to 40 and 41 to 60 are the majority of respondents. From the perspective of education level, respondents in general are less-educated, dominated by the level of primary and secondary schools. After six years of development in the study area, the number of residents with an average monthly income of more than 3,000 Yuan increases from 17.2% of the total population in 2010 to 41.4% in 2016, and the proportion of residents engaged in tourism is nearly 63%, accounting for the majority of the investigation objects. In general, the data from the two surveys is quite comparable.

### 3.2. Time Variation of Residents’ Perception of Impacts of Ecological Resettlement

#### 3.2.1. Dynamic Changes in the Perception of Economic Impacts

Compared with 2010, residents in resettlement locations in 2016 clearly support the items such as “increased commercial investment opportunities” (+0.67, *t* = 5.52 ***), “improved living standard” (+0.54, *t* = −3.84 **), “enhanced commodity economy consciousness” (+0.52, *t* = −3.79 **) and “changed consumption structure” (+0.61, *t* = −5.43 ***), etc. (Table 2). With a few notable exceptions, the perception intensity of two items decreases, namely, “increased economic benefits” (−0.26, *t* = 2.41) and “improved employment opportunities” (−0.25, *t* = 2.32). Residents believe that the improvement of their economic conditions is not directly related to the ecological resettlement project, but a natural result of local economic development in recent years. Therefore, residents’ perception of the positive impacts from above mentioned economic interest items are not significant. Under the background of in-depth development of the tourism industry, foreign investment has been increasing gradually, and residents have more opportunities to engage in local tourism development. Respondents have promoted the perception intensity of the item “increased rural outward labors” (+0.79, *t* = −6.56 ***), which reflects the number of migrant worker decreases in 2016. Due to the advantages of being close to home, low work intensity and relatively high income, residents in the resettlement locations are more inclined to participate in local tourism operations.

#### 3.2.2. Dynamic Changes in the Perception of Socio-Cultural Impacts

Residents in resettlement locations have significant changes in the perception of socio-cultural impacts of ecological relocation. In 2016, respondents strongly oppose the item “increased opportunities for trainings in science and technology” (−1.07, *t* = 8.88 ***). Due to limited service consciousness and managerial skills, most residents of Gaoyun express that they can generally only find low-tech and low-income jobs as hotel attendants, motorcycle taxi drivers and sanitation workers. Resettlers in Yuanjiajie and Tianzishan admit that the authorities provide more science and technology training opportunities, which is considered of little pertinence and practicability. Respondents agree on the items “more convenient access to external information” (+0.65, *t* = −4.48 ***) and “expanded social circles” (+0.27, *t* = −2.68 *), etc. Among them, the Tianzishan community is seated in a relatively remote area, which makes it inaccessible to tourists in the initial stage of resettlement. With the popularization and application of Internet, residents begin to deal with tourists more frequently by posting information, such as housing supply and special agricultural products on the Internet. There is a significant change in the perception differences of the item “worse neighborhood than before” (+1.14, *t* = −9.26 ***). Residents consider that in 2010, the competition between family hotels and tourist shops was extremely fierce, and the contradictions of attracting customers and snatching business seriously affected neighborhood relations. In recent years, government subsidies, dividends and external investment have significantly increased, respondents tending to stick together to protect their own interests. As a result, neighborhood relations become relatively harmonious. Residents strongly support the item of “traditional values came under attack” (−0.95, *t* = 8.32 ***). Residents gradually disagree with hard work to make a living, but they agree with the idea of making a living through relocation projects. It can be seen that the ecological resettlement has influenced the lifestyles and values of the resettlers to a certain extent.

#### 3.2.3. Dynamic Change of the Perception of Resource-Environment Impacts

Compared with 2010, residents show more recognition in the positive impact of ecological resettlement with regard to tourism resources and environment, such as “natural eco-environment recovered” (+0.38, *t* = −3.54 *), “infrastructure conditions improved” (+0.41, *t* = −3.89 **) and “public service facilities increased” (+0.45, *t* = −4.18 **), etc. Resettlers in Tianzishan and Yuanjiajie can enjoy free services on eco-friendly cars of the scenic area, which make traffic more convenient than before; Gaoyun belongs to the unified resettlement, where installation of street lamps, road repairs, water and electricity supply and other measures have significantly improved the living conditions of the community. Respondents have a relatively lower perception of the item “local sanitary conditions improved” (−0.63, *t* = 5.56 ***), which is mainly due to inefficient disposal of rubbish, wastewater and illegal buildings in the resettlement locations. Respondents have a weakened perception of the item “social instability factors increased” (+0.56, *t* = −3.92 **). Tracing its root to the improvement of community management and public security maintenance in resettlement sites, as well as living subsidies provided to local residents, the motivation for theft, robbery and other crimes have been significantly reduced.

#### 3.2.4. Dynamic Change of the Perception of Psychological Impacts

Residents’ perception of the psychological impacts of ecological resettlement shows a weakening trend. During the study period, residents have a weakened perception of the items such as “gained respect in the new resettlement community” (−0.48, *t* = 4.56 **) and “transforming farmers into non-farmers” (−0.27, *t* = 2.51 *). The new resettlers receive a certain amount of living subsidies, by which their basic living needs can be satisfied even if they do not work, thus leading to the dissatisfaction of the local indigenous people, who are not receiving any payments. The original residents of the Tianzishan community can expand the buildings freely in their former residence. However, the living space is strictly restricted after they arrive at resettlement communities; hence they are unfriendly to the immigrants. Respondents consider that, after being relocated, they have to fulfill their corresponding obligations instead of enjoying the rights and interests brought by urban household registration. From this majority of respondents do not fully agree with the item of “transforming farmers into non-farmers” after resettlement. The respondents’ perception of the item “poor adaptation to new productive lifestyles” (+0.66, *t* = −5.52 ***) and “weak belongingness to new community” (+0.33, *t* = −2.96 *) is relatively weak. As of 2016, 17 years have passed since the first ecological resettlement project was implemented in the Wulingyuan scenic area, where most of the resettlers deem they have adapted to new productive lifestyles in current communities.

#### 3.2.5. Dynamic Change of Perception of Resettlement Policy Impacts

In 2016, the respondents’ perception of the item “follow-up supportive measures are imperfect” (+1.08, *t* = 9.91 ***) is relatively weak. The reason is that local authorities pay medical and social insurance premiums for the resettlers every year, and provide them with a minimum living allowance, which helps to create better production and living conditions for them. In 2016, residents’ perception of the item “resettlement security system is inadequate” (+0.55, *t* = −3.90 **) tends to weaken. Yet, they generally agree with the item “lack of supervision on policy implementation” (−0.56, *t* = 6.23 **), which mainly results from the limitation in publicity of government information and the civilians’ right to know. For instance, the majority of respondents have little idea about the specifics of purchasing insurance and the composition of monthly resettlement subsidies. Therefore, administrators should encourage local residents to participate in community management and attach importance to the construction of the rights and interests supervision system. Resettlers boost their perception of the item “resettlement policies neglect residents’ interests” (−0.34, *t* = 3.01 *), and believe that the ultimate aim of the ecological resettlement is not to protect the ecology of the heritage site, which directly affects their understanding and support on the resettlement policies. Residents’ perception is a crucial part of appraising the performance of resettlement policies. It is necessary and urgent to effectively guide the public opinion of the communities in order to ensure their recognition and support for subsequent resettlement project in Wulingyuan.

### 3.3. Differences in the Impact of Demographic Characteristics on the Perceptions

Based on the independent sample *t*-test of useable questionnaires and their corresponding data, this study analyses the perception difference of each item through the significance level of the research results. Then, using the one-way ANOVA, this paper analyzes the differences in the perceptions of economic, socio-cultural, resource and environment, psychological and resettlement policy impacts in view of the demographic characteristics of residents.

In Table 3, the *F* value describing the impact of resettlement on the economy as perceived by gender is 5.497 (*p* < 0.05) in 2010 and 1.986 (*p* < 0.05) in 2016. Males reported higher mean values than females for overall perception of economic impacts, resource and environment impacts and also resettlement policy impacts [44,45,46]. The F value of gender impact on psychological impact is 1.351 (*p* < 0.05) and 2.757 (*p* < 0.001) individually. The average female perception of psychological impact is higher than that of male, which confirms that women are less adaptable to the new productive lifestyles [47,48].

During the study period, age does not affect the economy, but people of different ages may have different views on the economic impact. The F value of age’s influence on economy is 23.029 (*p* < 0.01) and 10.511 (*p* < 0.001) severally. Residents aged from 21 to 40 and 41 to 60 years old have the strongest perception of economic impacts, which results from the fact that young and middle-aged people are the main participants in economic activities and they are relatively sensitive to the dynamic trend of community economic development. The F value of the influence of age on psychology is 4.204 (*p* < 0.01) and 3.543 (*p* < 0.001) respectively. The elderly have the strongest perception of psychological impacts, especially the negative perception of the item “transforming farmers into non-farmers”. The main reason is that the sense of security and belongingness of the aged depends on the land. Compared with 2010, there is no significant difference in the perceptions of socio-cultural, resource-environment impacts among respondents of different ages in 2016.

Compared with 2016, residents with different levels of education have an obvious dissimilarity in their perception of economic, socio-cultural, resource-environment, psychological and resettlement policy impacts (*p* < 0.01) in 2010. Through a study of the reasons, resettlers who engage in tourism industry account for the majority in both periods, mainly go in for low-skilled jobs. The average perceptions of the five indicators of those surveyed with junior high school are relatively low. This is due to the fact that the jobs they do are similar to those of residents with low education level, which generates their passive attitude perception.

In 2010 and 2016, the F value of average monthly income’s impact on the economy is 13.618 (*p* < 0.01) and 2.320 (*p* < 0.01) individually. A considerable proportion of residents in the resettlement locations previously engaged in tourism operational activities such as hotels, restaurants and tourist shops, consequently gaining lower salaries is relatively higher. Economic benefits were directly affected in the process of relocation, and thus their perception of economic impacts gradually tends to be negative. The F value of average monthly income’s impact on psychology is 2.234 (*p* < 0.05) and 2.318 (*p* < 0.001). Respondents whose monthly average income is more than 3001 Yuan have the highest perception of psychological impacts. In other words, the resettlers with higher monthly average income have gained more economic benefits from relocation. As a result, they are pleased with the ecological resettlement project. Among them, the perceptions of socio-cultural, resource and environment and resettlement policy impacts fail to be tested the t-value.

The residents who are involved in tourism have a strong perception of the impacts of economic and socio-cultural impacts. In 2010 and 2016, the F value of the economic impact from resettlers’ engagement in tourism is 1.570 and 0.707; The F value of socio-cultural impacts is 2.533 (*p* < 0.01) and 1.558 (*p* < 0.01) respectively. With the massive influx of tourists, the resettlers’ economic gains and employment opportunities significantly increased and their living standards have been gradually improving.

During the study period, the F value of economic impacts of the resettlement modes is 18.446 (*p* < 0.001) and 7.258 (*p* < 0.001) individually. In 2016, the average of concealed resettlement residents’ perception of economic impacts is evidently higher than in 2010. They still live in Wulingyuan with priority to employment in the nature-based tourism sector. This finding is largely attributed to the improvement of traffic accessibility in the Tianzishan community, and the increase of tourists bringing considerable tourism revenue. Respondents of the Yuanjiajie community have the most positive perception of economic impacts and relocation policy impacts. Nevertheless, due to the lack of follow-up supportive measures for production, residents of Gaoyun community have relatively weak perception of economic impacts.

### 3.4. Regression Analysis of Residents’ Overall Perceptions in 2010 and in 2016

Stepwise multiple regressions are employed to analyze the overall perceptions of residents in resettlement locations in 2010 and 2016. As shown in Table 4, in 2010, the tolerance of independent variables ranged from 0.562 to 0.968 and the Variance Inflation Factor (VIF) value was 1.153–1.779; whereas in 2016, the tolerance of independent variables ranged from 0.435 to 0.927 and the VIF value was 1.079–2.297.

In 2010, thirteen of the forty-one independent variables reflect the overall satisfaction of respondents with the resettlement project. Respondents have the strongest perceptions of economic impacts and relocation policy impacts. In terms of perception of relocation policy impacts, residents receive narrow benefits from resettlement project, which appeals to the improvement and promotion of resettlement policies to a certain extent. From the perspective of perception of economic impacts, respondents acquire more employment opportunities and elevate the living standards after being relocated. Demographic characteristics such as educational level and modes of resettlement also have significant impact on the overall perceptions, among which respondents with higher educational level tend to have a relatively low satisfaction with ecological resettlement.

In 2016, ten of the forty-one independent variables explain the overall perceptions of the residents in the resettlement. Variation in residents’ perceptions of economic impacts and relocation policy impacts are greater than those of the other variables and become the dominating factor of residents’ overall perceptions. Due to the influx of outsiders and their low level of vocational skills, respondents face the severe pressure of modern society, such as intense employment pressure and psychological pressure. There are some reasons for respondents’ lower satisfaction with resettlement projects, for instance, the safeguard system for relocation is evidently imperfect and relocation policies only benefit minorities. The development of the offspring in the resettlement locations also affects the residents’ evaluation of the overall perceptions. Owing to the low education level and weak livelihood ability, they are anxious about whether the welfare policy can benefit descendants.

Therefore, the issues of employment and children’s education should be the focus of the follow-up ecological resettlement in the Wulingyuan scenic area.

## 4. Discussion

Previous literature has examined the ecological resettlement’s intensive influence on residents’ life satisfaction. However, the residents’ diachronic perception of the impacts of resettlement has not received much attention from scholars. It is important to note that from the Chinese authorities perspective, the local communities movement to new settlements envision the Geosystem’s balance rehabilitation [49,50]. For this reason, our research purveys a new insight into the perception of impacts of resettlement and paves the road for future studies in this field.

In this article, we systematically analyzed the differences in the residents’ perception of impacts of economic in the Wulingyuan scenic area. Residents experienced the greatest dissimilarity in the items such as “increased commercial investment opportunities”, “changed consumption structure” and “increased rural outward labors”. Tourism development increased the opportunities of commercial investment to residents, which is similar to the findings by Andereck et al. [10]. It is also accompanied by an apparent augmentation in the price level and a promotion in foreign investments, which boost the cost of living expenses for residents and reduce employment. Consequently, residents’ perception of impacts of economic has declined, which is in accordance with the findings of Saveriades [40]. In order to advance with the understanding of the impacts of resettlement in heritage resorts, this study adds three kinds of residents’ perception of economic impacts of different genders, age groups, education levels and incomes under the relocation mode. Compared with 2016, the residents of resettlement areas with different gender, age and monthly average income level have a remarkable impact on the perception of economic impacts. Residents who invest in the industrial resettlement mode have a relatively positive perception of the economic impacts. It can be seen that the perception of economic impacts is the main factor affecting residents’ overall perceptions of resettlement.

From the perspective of social and cultural influences, respondents have a neutral attitude towards the social and cultural impacts brought by ecological relocation, indicating that the ecological resettlement has no intensive influence on local culture. As far as the subdivided indicators are concerned, residents’ perceptions of science and education training, information acquisition, neighborhood relations and traditional values are unique, which is consistent with the research conclusion of Fernando et al. [16], who demonstrated that social and cultural influences display a wide range of perceptions, including lifestyle habits, humanities, social life, beliefs and values. Residents with disparate demographic characteristics have different perceptions of social and cultural effects. For instance, residents who received better education have a more comprehensive understanding of the overall implementation of ecological relocation, and they pay more attention to the socio-cultural impacts of ecological resettlement to residents, and lower education. Residents have a negative perception of socio-cultural impacts.

From the perspective of resources and environment, it mainly refers to the impacts of ecological resettlement on the resources and environment of the scenic areas and the life space. The respondents generally are in favor of the positive impacts of ecological resettlement with regard to the resources and environment, and fully recognize the ecological relocation in the scenic spot. The role played by natural ecological restoration, infrastructure and public service facilities, is in line with other researchers findings [17,51]. Simultaneously, it also verifies the residents’ negative perception of impacts of ecological environment conducted by Yoon et al. [52] from the aspect of negative impact of resources and environment. The research results show that compared with 2010, residents of Tianzishan have a more negative perception of the environmental health of the scenic spot in 2016, and residents of Gaoyun have a higher degree of recognition of the improvement of public service facilities. The Yuanjiajie respondents have the most different perception of this indicator.

At the impacts of psychological and resettlement policy level, the residents’ perception of psychological impacts shows a weakening trend, while the cost perception displays a strengthening trend, namely, residents are more inclined to perceive the benefits brought by resettlement. As an illustration, residents will get a certain living allowance after moving which can satisfy their living standards even if they remain unemployed. Hence, after moving to a new community, the residents of the original place of residence will be dissatisfied comparing the two living conditions. The resettlement policy is related to the direct interests of the residents and the future production and living security. The residents’ perception of resettlement policy impacts remains negative. Although the residents have higher recognition of relocation, the residents’ interest expectations on the resettlement policy have not been satisfied. It is embodied in the fact that it is more empathetic to items such as “traditional values came under attack” and “lack of supervision on policy implementation”.

Our research makes important contributions to the literature researching the ecological resettlement. At the level of theoretical research, this study establishes a model that is applied to appraise the perception of the impacts of ecological resettlement in the Wulingyuan scenic area, providing a reference for future similar researches. In response to the lacunae related to the evaluation of resettlement policy based on the cross-sectional analysis, our study explores the residents’ perception in 2010 and 2016 regarding the ecological relocation, which will deepen and enrich the content of ecological resettlement.

There are several limitations in this article though they prepare the base for future studies. Further research might address the perception of other stakeholders on ecological resettlement, in order to understand and assess the implementation effects of relocation processes more comprehensively.

## 5. Conclusions

The empirical results are presented as follow. Firstly, during the study period, the residents in the resettlement locations were more positive in terms of resource-environment impacts, economic impacts, socio-cultural impacts, psychological impacts, and their perception of resettlement policies were relatively negative. Secondly, compared to 2010, the residents’ perceptions of impacts (economic, psychological, according to different genders, ages and monthly average income levels) are more significant in 2016. Thirdly, during six years, the perceptions of impacts of economic and resettlement policy have become the dominant factors affecting the overall perception of relocation, and the issues of employment, children’s education and resettlement security system are the focus of continuous attention of our respondents.

Based on the aforementioned conclusion, this article proposes the following policy recommendations for promoting sustainable development of world heritage sites. Firstly, the authorities should make the ecological resettlement projects and their progress public, enhancing the transparency of the relocation processes; regarding policy implementation, we should be honest, match words with deeds, establish prestige, regain the trust of the masses, and strengthen the supervision on the resettlement policy implementation. Secondly, the authorities should invest more in education in order to improve the overall education level of residents. It is necessary and urgent to strengthen the ideological education of resettlement communities, guide residents to look at the living conditions after relocation rationally, and encourage their rational investment and consumption. Thirdly, different ecological resettlement measures can be adopted for communities organizing cultural and recreational activities, encouraging residents to participate, strengthening communication and solidarity among locals.

This paper examined the main factors affecting the residents’ perception of ecological resettlement from the demographic and statistical characteristics of endogenous variables. It has certain theoretical and practical significance to better understand the performance of the relocation project from the perspective of the residents’ perception. However, it is also crucial for the government, scenic area administration, neighborhood committees, investors, non-relocated residents and other stakeholders to evaluate the overall performance of ecological relocation.

## Figures and Tables

**Table 1:** Social demographic characteristics of the respondents in 2010 and 2016.

Item	Construction	2010		2016	
		Sample Size	Percentage (%)	Sample Size	Percentage (%)
Sex	Male	175	48.6	178	51.9
	Female	185	51.4	165	48.1
Age	≤20	12	3.3	15	4.4
	21–40	175	48.6	156	45.5
	41–60	127	35.3	131	38.2
	≥61	46	12.8	41	12.0
Net income per month (Yuan)	≤1000	107	29.7	96	28.0
	1001–2000	86	23.9	39	11.4
	2001–3000	105	29.2	66	19.2
	≥3001	62	17.2	142	41.4
Modes of resettlement	Tianzishan community	128	35.6	115	33.5
	Gaoyun community	121	33.6	109	31.8
	Yuanjiajie community	111	30.8	119	34.7
Engagement in tourism	Yes	225	62.5	217	
	No	135	37.5	136	36.7
Education level	Elementary school or lower	91	25.3	114	33.2
	Junior middle school	166	46.1	100	29.2
	High school	81	22.5	78	22.7
	Junior College or higher	22	6.1	51	14.9
Proportion of tourism receipts (%)	≤10	111	30.8	81	23.6
	11–40	74	20.6	96	28.0
	41–70	93	25.8	82	23.9
	≥71	82	22.8	84	24.5

**Table 2:** Independent sample *t*-test of residents’ perception of impacts of ecological resettlement.

Perception	Key Indicator	Mean Value of Perceptual Intensity		Change from Period 2010 to 2016	*t*
		2010	2016		
F1	Increased economic benefits	3.97	3.71	−0.26	+2.41
	Improved living standard	3.13	3.67	+0.54	−3.84 **
	Increased commercial investment opportunities	3.11	3.78	+0.67	5.52 ***
	Enhanced commodity economy consciousness	3.17	3.69	+0.52	−3.79 **
	Improved employment opportunities	3.68	3.43	−0.25	2.32
	Changed consumption structure	3.50	4.11	+0.61	−5.43 ***
	Enlarged rich-poor wealth gap	2.69	2.77	+0.08	−1.57
	Increased rural outward labors	2.90	3.69	+0.79	−6.56 ***
F2	Increased opportunities for cultural and recreational activities	3.32	3.10	−0.22	2.01
	Increased opportunities for trainings in science and technology	3.40	2.33	−1.07	8.88 ***
	Improved school conditions for children	3.90	3.69	−0.21	1.93
	More convenient access to external information	3.61	4.26	+0.65	−4.48 ***
	Expanded social circles	3.57	3.84	+0.27	−2.68 *
	Worse neighborhood than before	2.75	3.89	+1.14	−9.26 ***
	Less access to the core scenic spots	3.14	3.20	+0.06	−1.26
	Ancient folk customs faded	2.73	2.72	−0.01	0.36
	Traditional values came under attack	2.91	1.96	−0.95	8.32 ***
	Debasement of moral standards	2.95	2.71	−0.24	2.29
F3	Natural eco-environment recovered	4.07	4.45	+0.38	−3.54 *
	Tourism landscape resources effectively protected	4.18	4.39	+0.21	−1.76
	Local sanitary conditions improved	4.35	3.72	−0.63	5.56 ***
	Public service facilities increased	3.70	4.15	+0.45	−4.18 **
	Infrastructure conditions improved	4.01	4.42	+0.41	−3.89 **
	Social instability factors increased	3.03	3.59	+0.56	−3.92 **
F4	Gained respect in the new resettlement community	3.46	2.98	−0.48	4.56 **
	Transforming farmers into non-farmers	2.74	2.47	−0.27	2.51 *
	Poor adaptation to new productive lifestyles	3.27	3.93	+0.66	−5.52 ***
	Weak belongingness to new community	3.25	3.58	+0.33	−2.96 *
	Sore pining for the original residence	3.27	3.04	−0.23	2.18
F5	Resettlement policies neglect residents’ interests	2.34	2.00	−0.34	3.01 *
	Resettlement policies only benefit minorities.	2.57	2.37	−0.20	1.71
	Resettlement security system is inadequate	1.58	2.13	+0.55	−3.90 **
	Follow-up supportive measures are imperfect	1.62	2.70	+1.08	9.91 ***
	Lack of supervision on policy implementation	2.20	1.64	−0.56	6.23 **
F6	Overall satisfaction with the resettlement	3.08	3.03	−0.05	1.24

F1: Perception of economic impacts; F2: Perception of socio-cultural impacts; F3: Perception of resource-environment impacts; F4: Perception of psychological impacts; F5: Perception of resettlement policy; F6: Overall perceptions. * *p* < 0.05; ** *p* < 0.01; *** *p* < 0.001.

**Table 3:** Demographic profiles of factors on perception of ecological resettlement.

Demographic Factor		Mean Value of Perceptual Intensity									
		F1		F2		F3		F4		F5	
		2010	2016	2010	2016	2010	2016	2010	2016	2010	2016
Sex	Male	3.85	3.75	3.21	3.20	3.88	4.12	3.11	3.15	3.86	2.20
	Female	3.72	3.47	3.26	3.14	3.87	4.11	3.30	3.25	3.83	2.14
	*F*	5.497 *	1.986 *	0.428	0.521	0.288	1.241	1.351 *	2.757 ***	0.231	1.217
Age	≤20	2.97	3.57	3.60	3.27	3.46	4.28	2.74	3.52	3.43	2.05
	21–40	3.30	3.85	3.57	3.23	3.77	4.08	2.86	3.15	3.42	2.19
	41–60	3.14	3.69	2.92	3.21	3.97	4.15	3.33	3.10	3.41	2.19
	≥61	2.88	3.38	2.63	2.79	4.16	4.11	3.58	3.63	3.52	2.03
	*F*	23.029 ***	10.511 **	3.55**	1.520	5.635 **	1.084	4.204 **	3.543 ***	0.564	0.835
Education level	Elementary school or lower	3.06	3.53	3.09	3.15	3.62	4.19	3.08	3.20	3.60	2.07
	Junior middle school	3.12	3.74	3.17	3.16	3.70	4.04	3.24	3.25	3.72	2.31
	High school	3.18	3.68	3.44	3.24	3.75	4.12	2.93	3.18	3.81	2.27
	Junior College or higher	3.38	3.40	3.61	2.98	4.02	4.11	3.05	3.13	3.55	1.96
	*F*	5.476 **	1.213	4.204 **	2.221	3.352 **	0.951	2.835 **	1.329	3.203 **	0.736
Net income per month (Yuan)	≤1000	3.51	3.49	3.16	3.09	3.87	4.17	3.04	3.37	3.86	2.08
	1001–2000	3.52	3.49	3.35	3.27	3.88	4.34	3.14	3.33	3.87	2.10
	2001–3000	3.40	3.67	3.32	3.22	3.86	4.01	3.06	3.06	3.88	2.16
	≥3001	2.90	3.68	3.27	3.17	3.88	4.08	3.19	3.11	3.90	2.25
	*F*	13.618 **	2.320 **	2.236 *	9.810	0.879	1.196	2.234 *	2.318 ***	1.896 *	1.149
Engagement in tourism	No	2.86	3.46	3.10	3.10	3.85	4.13	3.17	3.19	3.76	2.01
	Yes	3.01	3.69	3.29	3.21	3.89	4.12	3.08	3.21	3.84	2.26
	*F*	0.707*	1.570 *	2.533 *	1.558 *	0.123	0.993	2.235 *	1.004	2.654 *	1.111
Proportion of tourism receipts (%)	≤10	3.13	3.40	3.10	3.12	3.87	4.18	3.18	3.24	3.79	2.00
	11–40	3.21	3.69	3.34	3.14	3.87	4.14	2.99	3.19	3.84	2.33
	41–70	3.12	3.60	3.32	3.17	3.54	4.15	3.08	3.25	3.94	2.26
	≥71	3.01	3.84	3.24	3.30	3.92	3.97	3.11	3.14	3.84	2.15
	*F*	3.444 *	1.613	3.290 *	2.388	1.696	0.946	2.749	1.367 *	0.657	1.012
Modes of resettlement	Tianzishan community	3.05	3.89	3.17	3.17	3.86	3.78	3.06	3.10	3.87	2.10
	Gaoyun community	3.29	2.77	3.45	3.10	3.90	4.19	3.66	3.23	3.84	1.50
	Yuanjiajie community	3.24	4.11	3.37	3.14	3.89	4.14	3.61	3.27	3.81	2.83
	*F*	18.446 ***	7.258 **	3.875 **	1.159	0.523	2.247 *	5.279 **	1.210	0.421	6.578 **

**Table 4:** Stepwise multiple regressions analysis of residents’ overall perceptions.

Year	Variable	Standard regression Coefficient β	*t*	Accumulative Contribution Rate	Collinearity Diagnosis	
					Tolerance	Variance Inflation Factor (VIF)
2010	Resettlement security system is inadequate	0.460	12.662 ***	0.396	0.718	1.393
	Improved employment opportunities	0.171	4.791 ***	0.476	0.745	1.342
	Resettlement policies neglect residents’ interests	0.178	4.710 ***	0.531	0.663	1.508
	Resettlement policies only benefit minorities.	0.150	3.642 ***	0.561	0.562	1.779
	Education level	−0.230	−6.715***	0.585	0.808	1.237
	Enlarged rich-poor wealth gap	0.139	3.557 ***	0.601	0.617	1.621
	Local sanitary conditions improved	−0.204	−5.673 ***	0.614	0.734	1.362
	Increased opportunities for cultural and recreational activities	0.130	3.779 ***	0.630	0.797	1.255
	Public service facilities increased	0.104	3.136 ***	0.642	0.868	1.153
	Modes of resettlement	0.135	3.524 ***	0.652	0.650	1.540
	Social instability factors increased	−0.104	−3.042 ***	0.659	0.818	1.223
	Changed consumption structure	0.088	2.620 ***	0.666	0.849	1.177
	Improved school conditions for children	0.089	2.353 *	0.672	0.661	1.514
2016	Improved employment opportunities	−0.231	−3.824 ***	0.257	0.599	1.668
	Improved living standard	−0.153	−2.755 ***	0.346	0.709	1.410
	Lack of supervision on policy implementation	1.520	2.806 ***	0.364	0.824	1.265
	Infrastructure conditions improved	0.141	2.815 ***	0.372	0.878	1.138
	Improved school conditions for children	−0.185	−3.765***	0.381	0.907	1.102
	Net income per month(Yuan)	0.137	2.817 ***	0.396	0.927	1.079
	Modes of resettlement	−0.233	−3.290 ***	0.413	0.435	2.297
	Traditional values came under attack	1.350	2.792 ***	0.428	0.568	1.952
	Resettlement security system is inadequate	1.240	2.159 *	0.437	0.758	1.302
	Increased opportunities for trainings in science and technology	−0.120	−2.029 *	0.446	0.622	1.609
